# The effects of personality and locus of control on trust in humans versus artificial intelligence

**DOI:** 10.1016/j.heliyon.2020.e04572

**Published:** 2020-08-28

**Authors:** Navya Nishith Sharan, Daniela Maria Romano

**Affiliations:** a Department of Psychology, University College London, London, WC1E 6BT, UK; b Department of Information Science, University College London, London, WC1E 6BT, UK

**Keywords:** Psychology, Trust, Artificial intelligence, Locus of control, Big five personality traits, Individual traits

## Abstract

**Introduction:**

We are increasingly exposed to applications that embed some sort of artificial intelligence (AI) algorithm, and there is a general belief that people trust any AI-based product or service without question. This study investigated the effect of personality characteristics (Big Five Inventory (BFI) traits and locus of control (LOC)) on trust behaviour, and the extent to which people trust the advice from an AI-based algorithm, more than humans, in a decision-making card game.

**Method:**

One hundred and seventy-one adult volunteers decided whether the final covered card, in a five-card sequence over ten trials, had a higher/lower number than the second-to-last card. They either received no suggestion (control), recommendations from what they were told were previous participants (humans), or an AI-based algorithm (AI). Trust behaviour was measured as response time and concordance (number of participants' responses that were the same as the suggestion), and trust beliefs were measured as self-reported trust ratings.

**Results:**

It was found that LOC influences trust concordance and trust ratings, which are correlated. In particular, LOC negatively predicted beyond the BFI dimensions trust concordance. As LOC levels increased, people were less likely to follow suggestions from both humans or AI. Neuroticism negatively predicted trust ratings. Openness predicted reaction time, but only for suggestions from previous participants. However, people chose the AI suggestions more than those from humans, and self-reported that they believed such recommendations more.

**Conclusions:**

The results indicate that LOC accounts for a significant variance for trust concordance and trust ratings, predicting beyond BFI traits, and affects the way people select whom they trust whether humans or AI. These findings also support the AI-based algorithm appreciation.

## 1 Introduction

Trust is a complex, multifaceted construct that is an essential aspect of interpersonal relationships (Simpson, 2007). People rely on trust to guide their behaviour when they cannot rely on societal norms and their cognitive resources to make an informed, rational decision (Thagard, 2018). Trust in technology, explicitly, is the belief that the system will do what it is expected to do (Li et al., 2008).

We are increasingly, and often seamlessly, exposed to highly specialised artificial intelligent (AI) based algorithms. While some people trust AI-based algorithms to provide suggestions for everyday decisions in their lives (e.g., suggesting a purchase, directing them home, or summarising the status of their fitness), others lack trust in AI-based technologies (Davenport, 2018). A survey (Krogue, 2017) presented respondents with a list of AI-based services and found that 41.5% of respondents did not trust any of the given resources. The same survey illustrated the low adoption of AI-based services in professional sectors with only 9% trusting financial AI services and only 4% trusting AI-based services with hiring employees. Abbass et al. (2016), propose a generalised model of trust for humans and machines, where trust is a social operator that balances the complexity inherent in social systems and the environment. According to Abbass (2019), it might be possible to standardise all AI-based services by adding an interface that assesses the human-trustworthiness and presents people with the information at a sufficient pace and in a form suitable for them to understand and act. This claim appears to assume that that trust depends on personality traits, which influence both trust in other people as well as AI-based services. Therefore, we decided to investigate the trust in humans versus the trust in an AI-based algorithm from a psychological perspective.

### 1.1 Interpersonal trust and measures

In our everyday life, trust mediates the human-human interactions and shapes our relationships by increasing our security in them and reducing inhibitions and defensiveness (Larzelere and Huston, 1980). Human trust is studied from the point of view of various disciplines. For example, Krueger and Meyer-Lindenberg (2019) propose a model of interpersonal trust combining complementary methodologies in economics, psychology, and neuroscience. Despite the perspectives of the different disciplines, according to Thielmann and Hilbig (2015), scholars agree on the essential components of trust. Based on their extensive literature review they define interpersonal trust as "*a risky choice of making oneself dependent on the actions of another in a situation of uncertainty, based upon some expectation of whether the other will act in a benevolent fashion despite an opportunity to betray*” (Thielmann and Hilbig, 2015, p 251). This definition includes all aspects they have identified in the literature. It separates trust cognition (e.g. expectations), from trust behaviour (e.g. choice made), from the willingness to trust, suggesting that those can be separately assessed (observed and quantified). In particular, they describe the willingness to trust as a psychological state.

Ben-Ner and Halldorsson (2007) have reviewed the literature in search of empirical measures of trust, and report that researchers either investigate the behaviour that highlights different degrees of trust or directly survey people on whether (or to what degree) they trust others. They found that only a limited number of studies utilise and relate both measures. They suggest that utilising behavioural measures together with self-reported answers capture the different dimensions of trust, which can be further complemented with the attitude towards risk (willingness to be vulnerable) and belief in others' honesty (having a positive perspective on the intentions of others). Bauer and Freitag (2018) divide trust as an expectation, from the behaviourally exhibited trust, highlighting that trust is situation-specific, and can be described with the following parameters "*a truster A that trusts (judges the trustworthiness of) a trustee B with regard to some behavior X in context Y at time t* “(Bauer and Freitag, 2018, p 2). They state that adding time to the parameters defining trust highlights that trust can change over time as the person adapts his/her expectations over time. However, independently of the parameters, they believe that some people are more trusting than others. In literature, de Visser et al. (2012) highlights that while breaches of trust from humans are easy to forgive, trust in automation, once lost, is difficult to regain. This suggests that it is necessary to find a manner to measure trust over time, and with components that can be measured both for trust in humans and AI. In particular, Chancey et al. (2015) measured reaction time, agreement rate, and the subjective estimates of trust in a signalling system, and found that the subjective estimate of trust does not affect response behaviour, suggesting that the concept of trust in automation might be determined by the multiple variables besides self-reported trust.

In this section, we have reviewed the concept of interpersonal trust and the parameters that can help define it. In particular in search of measures that can be used for both interpersonal trust (human-human) and human-AI trust the literature suggests investigating both behavioural measures (e.g. response time, agreement rate or concordance with suggestion) as well as self-reported trust and how these change over time.

### 1.2 Trust in humans and AI

In this paper, the terms AI or automation are used interchangeably to describe *AI-based algorithms that autonomously execute a task to aid human-decision making*. The AI considered does not act autonomously, but rather present the results to the human, who might, or not, consider such suggestions. Also, the AI in question does not exhibit any particular human-like behaviour or visual features. In literature, trust in technology is seen often as dependent on the degree of anthropomorphism of the technology. For example, Waytz, A., Heafner, J., & Epley, N. (2014) investigated people's willingness to trust technology in the context of autonomous vehicles and found that participants trusted that the vehicle would perform more competently as it acquired more anthropomorphic features.

Similarly, de Visser, E. J., Monfort, S. S., McKendrick, R., Smith, M. A., McKnight, P. E., Krueger, F., & Parasuraman, R. (2016) found that anthropomorphism increases trust resilience in cognitive agents. Finally, for robots, Sanders et al. (2011) report that, amongst other factors, robots’ ability to express social behaviours (e.g., turn-taking, emotional expressions) and anthropomorphism, are correlated with trust. Thus, in the AI considered in this research, we have eliminated any human-like element (behavioural or visual), which could be investigated in future research.

Currently, we trust the suggestions of some AI-based algorithms that are considered reliable, although they exhibit a high level of autonomy when computing results, as they provide a service in the best interest of the human using them. Examples of commonly used technologies are the navigator planner in our car (or phone), or the in-car collision avoidance system, which are quickly making their way into the most recent cars (e.g., Honda Accord, Toyota Prius, Madza CX-5) and they can sense danger and react faster than humans. According to Abbass (2019), these are just AI-based autonomous functions that still leave the user feeling significantly in control. Yet, not everyone might feel comfortable even with these standard AI-based services from the start. For example, Al Mahmud, Mubin and Shahid (2009) state that younger drivers have a more positive user-experience than older ones when using a new navigation system; while Samson and Sumi (2019) emphasise that navigation systems need to be personalised to encourage their use in all situations.

Literature shows that people trust automation (or AI-based autonomous functions) more than other humans. A study (Lewandowsky et al., 2000) compared to trust in automation with trust in human partners in a process control task, in which they delegated specific sub-tasks to 'auxiliary' controllers. The participants in Lewandowsky et al. (2000) experiment were either told that the auxiliary controllers were other participants or algorithms, and faults were introduced at different points in the task to observe its effects on the trust dynamic and participants' self-confidence. They found that while participants could not avert or correct the effects of the faults, they could choose not to delegate, and they delegated sub-tasks to automation more than human partners, where trust and self-confidence predicted this reliance. Lewandowsky et al. (2000) study appear to indicate that not only do people trust automation more than humans but also that trust in automation is especially displayed when they cannot rely on their own judgement.

Newer studies also support this notion of algorithm appreciation. Logg, Minson and Moore (2019) conducted experiments comparing people's adherence to advice when they thought it was coming from an algorithm, with when they thought it was coming from a person. In the study, participants completed tasks in which they made quantitative judgements and received advice. The source of the advice was manipulated (either human or algorithm) to measure participants' preferences. Logg et al. (2019) found that, in general, people undervalue advice. However, laypeople were more reliant on advice when they thought it was coming from an algorithm than other humans. The effect faded when experts had to choose between the algorithm and their judgement.

Hoff and Bashir (2015) performed a systematic review of the factors that influence trust in automation, and present a three-layered trust model which include dispositional, situational and learnt trust. They continue that dispositional is the most stable of the three levels and is defined as: “*Dispositional trust represents an individual's overall tendency to trust automation, independent of context or a specific system, … (due) to long-term tendencies arising from both biological and environmental influences.*” (page 413, Hoff and Bashir (2015)). They continue stating that dispositional trust has four primary sources of variability in this most basic layer of trust: culture, age, gender, and personality. Schaefer et al. (2016), following an extensive meta-analysis review of the articles dealing specifically with human-automation trust, propose a three-factor model of trust in automation, highlighting that these are: environment-related (team collaboration, task/context), partner-related (partner's features and capabilities), human-related (traits, states, cognitive and emotive factors). In particular, they calculated the correlation effect size for each research, concluding that antecedents of trust in automation are age, gender, ethnicity and personality.

In this section, we have highlighted the differences found in the literature with regards to trust in humans and trust in AI, where there seems to be an algorithm appreciation especially when people do not feel expert in making the decision. In addition, human-related traits might also influence trust, in both humans and AI, as it will be reviewed in the following sections.

### 1.3 Personality and trust

The propensity to trust can be understood as a personality trait (Rotter, 1967). In a recent study Alarcon et al. (2018) analysed the impact of personality, measured using the Big Five model (e.g., De Raad (2000)), across the entire trust process (trusting actions or behaviour, beliefs and intentions), and found that personality predicted trust intentions and beliefs, but not trust behaviours; while propensity to trust predicted all three aspects of trust. Müller and Schwieren (2019) experimentally tested the impact of personality factors (measured using the Big Five model) on behaviour in a trust game among humans and found that (despite Alarcon et al. (2018) findings) personality traits do explain trust behaviour. However, they suggest that personality best correlates with behaviour in ambiguous decisions, and not well in a risky decision where trust behaviour is better explained also considering whether one is in a strong or a weak situation.

According to trait psychology, there are five major facets of human personality, openness, conscientiousness, extraversion, agreeableness, and neuroticism. The literature widely supports the idea that these factors, from the Big Five Inventory (BFI) of personality, explain individual differences in behaviours and social attitudes (e.g. John et al., 2008; McCrae, 2000). Table 1 briefly explains each of the dimensions.

**Table 1:** Big five personality dimensions and definitions.

Big Five Dimension	Definition
**Openness** – Closedness	Curious and seeking new experiences
**Conscientiousness** – Lack of direction	Organised, meticulous, and reliable
**Extraversion** – Introversion	Energetic, assertive, and seeking excitement
**Agreeableness** – Antagonism	Warm, friendly, and helpful
**Neuroticism** – Emotional stability	Anxious and moody

Considering the individual dimensions, Thielmann and Hilbig (2015) link interpersonal trust (human-human) to the basic personality traits, suggesting that Neuroticism and Agreeableness are the main two factors responsible for individual differences in trust behaviour. Also, Ben-Ner & Halldorsson (2007) found that participants’ personality predicts quite well interpersonal trust, where more agreeable and extroverted and less conscientious participants are more trusting. For human-AI trust, Chien et al. (2016) examined the effects of personality traits on trust in automation in participants from American, Taiwanese, and Turkish cultures. They found that personality differences significantly influenced trust in automation and that initial trust formation significantly correlates with agreeableness and conscientiousness, where participants who scored higher on agreeableness or conscientiousness trusted automation more.

Mooradian et al. (2006) circulated a self-administered questionnaire, which measured personality traits and interpersonal trust at work, among the employees of a company, and found a significant relationship between agreeableness and propensity to trust. A similar study by Jacques et al. (2009) investigated the effect of personality on trust in virtual reality (VR) teams, where, due to the use of VR technology, individuals were unable to control others’ behaviours and were forced to trust them. Their results indicated that extraversion and agreeableness negatively correlated with trusting behaviours. Neuroticism positively correlated with trust behaviour, and openness negatively correlated with technology anxiety.

In this section, we have reviewed how personality can explain trust behaviour in ambiguous decisions, but less well in risky decisions. Literature suggests individual dimensions of personality might explain trust in humans and in AI; however, personality might not explain trust behaviour completely. In addition, the situation and its predictability might play a role in trust. Thus, we have investigated a case in which it is difficult to predict the situation clearly and in which the AI is only randomly correct, as it will be explained in the following sections.

### 1.4 Locus of control, trust and decision making

Locus of control (LOC) is a person's belief about the causes of his/her experiences in an unpredictable situation (Duttweiler, 1984), where one attributes the responsibility of the situation to external factors (external or low LOC) or oneself (internal or high LOC) (Joelson, 2017). Conversely, people with an internal control feel that they are masters of their life and capable of influencing their environment. In contrast, those with an external locus of control believe that their ability to control their life is low.

LOC has been linked to decision making and use of information in literature, for example, Olaronke and Sunday (2015) have connected internal LOC with independent decision making and good use of information in aviation; Selart (2005) has shown that managers with external LOC tend to be more consultative in their decision, and more frequently use participative decision-making with other humans.

McCanne and Lotsof (1987) found that subjects that are more perceptually vigilant tend to have low interpersonal trust and an external locus of control. In more recent years, LOC has been connected with trust amongst humans in specific contexts. For example, Rodriguez-Ricardo et al. (2019) found that altruism and internal locus of control enhance trust in crowdfunding. Hillen, de Haes, Stalpers, Klinkenbijl, Eddes, Verdam & Smets (2014) found that locus of control influence patients' trust in their oncologist. Similarly, Gopinath et al. (2000) found, amongst South Indian patients, that those with an external locus of control have a reduced trust in the medical system, and thus compliance to the therapy. Triplett and Loh (2018) found that trust moderates the relationship between work locus of control and psychological safety in organisational work teams.

Lee-Kelley (2006) found that participants with an internal locus of control felt that time is needed in order to build trust amongst co-workers in a virtual team (humans geographically separated, but working on the same project). As such, LOC might influence trust in situations in which we deal with partners in technology-mediated environments.

In this section, we have seen that LOC is a trait that intervenes in situations of uncertainty, internal LOC leads to independent decision and good use of information, external LOC leads to a more consultative decision-making approach. However, the relationship between LOC, personality and trust, both interpersonal and with AI-based algorithms, is not widely investigated in literature and the object of this research.

## 2 Aims and hypotheses

As seen in the literature reviewed in the previous sections, trust in humans, or interpersonal trust, is best measured by both behavioural evidence and self-reported expectations (Ben-Ner and Halldorsson, 2007). Also, trust is situation-specific and can change over time (Bauer and Freitag, 2018). With regards to AI, when people lack experience, they tend to trust automation more than other humans (algorithm appreciation) (Lewandowsky et al., 2000; Logg et al., 2019). However, we appear to use automation in situations in which we still feel largely in control (Abbass, 2019). Personality does not explain trust behaviour fully; it best describes it in situations with no dangerous consequences (Müller and Schwieren, 2019), while in situations in which we feel unable to control others, the use of information and consultative decision-making approach might depend more on our locus of control (Selart, 2005).

In the present study, the task is deliberately chosen to be a card game, which puts the player in an ambiguous decision, rather than a risky one, with no particular consequence rather than entertainment. In this task, according to literature, personality traits could be a good predictor of trust behaviour. However, the behaviour of the agent providing the suggestion (human or AI) is not predictable (random) as such, the influence of LOC on trust is also tested.

This study investigates the effect of the *agent delivering the suggestion*, i.e., human or AI agent (independent variable), on trust behaviour measured as *reaction times*, and *concordance*, i.e., the extent to which participants follow the suggestions, and trust beliefs measured as self-reported *trust ratings* (dependent variables) in the context of a game-based decision task. A control condition was introduced, in which no suggestion was provided. Finally, to contextualise the findings (as observed through concordance and trust ratings) for the AI and human condition, self-reported trust was measured via a post-experiment questionnaire.

After completing questionnaires collecting data on their individual and personality traits, participants are randomly assigned to either the control, human or AI condition. They play a card game in which they are shown five cards with numbers. The last card is covered, and they need to decide whether the number of this card is higher or lower than the second-to-last card (for a more precise description see the Card Game section below). Participants in the human and AI conditions receive suggestions to help their judgement over ten trials. Reaction time and concordance are measured, and participants report how much they trusted the suggestions.

The following research question was formulated: to what extent do personality traits (Big Five traits) and LOC influence trust in humans and AI? Another subsidiary aim of this study was to validate the literature findings related to algorithm appreciation.

Based on previous studies, it is hypothesised that participants will trust AI more than humans and so more likely incorporate suggestions delivered by AI into their judgements responding faster. The detailed study hypotheses are as follows:

Hypothesis 1 BFI dimensions will predict the trust variables in both humans and AI conditions.

(In particular, we hypothesised that participants who score higher on extraversion, agreeableness, and openness, and low on neuroticism and conscientiousness, will have slower reaction time, and higher concordance and trust ratings in human and AI conditions).

Hypothesis 2 LOC will be positively related to trust beyond the BFI dimensions in both humans and AI conditions.

Hypothesis 3 The agent delivering the suggestion (Human or AI) affects trust.

(In particular, reaction times would be lowest for the AI, followed by the human condition and the control trials. Concordance and trust ratings would be higher for AI than the human condition).

## 3 Method

### 3.1 Participants

**Figure 1:**
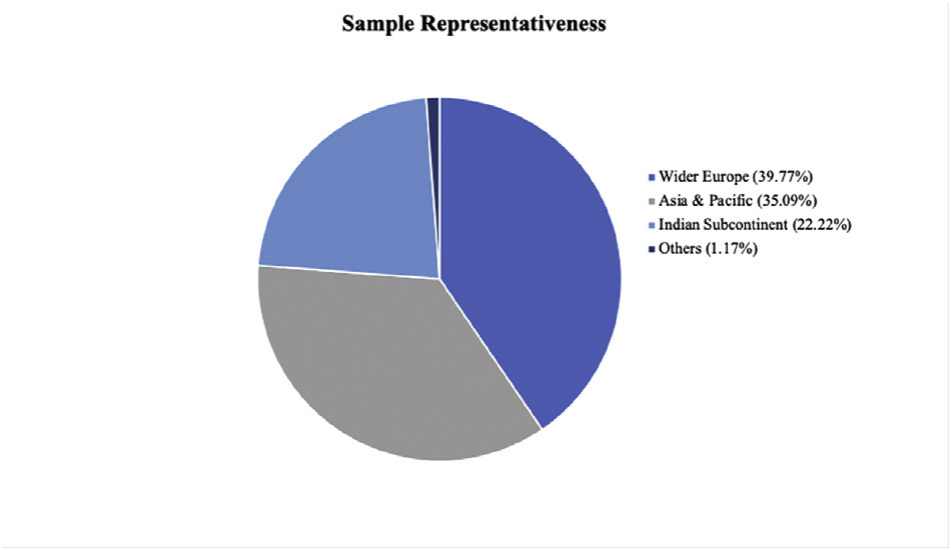
Percentage of participants represented in the sample (pie chart).

**Figure 2:**
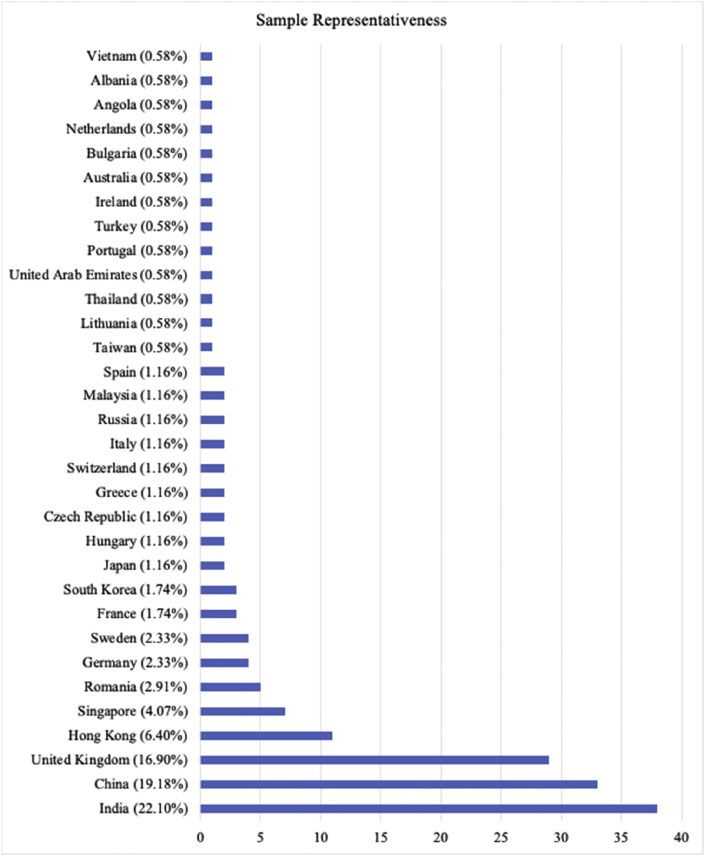
Percentage of participants in the sample from each of the countries (bar chart).

One hundred and seventy-one adult volunteers were recruited through the University Colege London (UCL) volunteers pool, social media (i.e., Facebook), and word of mouth and randomly assigned to one of three conditions, 58 to control, 57 to humans, and 57 to AI (one participant in the AI condition was discarded as the data was incomplete). Participants received no compensation for their participation. Among the valid data, 133 participants reported their gender as female, 37 as male, and one as other. Participants (Mean age = 22.6 years, *SE* age = 0.76 years, age range: 18–71 years) were from 32 different countries (see Figure 2), where the country was where they lived the most between the country of origin and the country of residence (Figures 1 and 2).

### 3.2 Software and display hardware used

The information, consent, task instructions, pre-task and post-task questionnaires, card-game, and debriefing, were designed and executed using Gorilla (https://gorilla.sc/), an online software that supports experiment design and data collection. While the experiment was online, due to the nature of its interface, access was restricted to ensure that it was completed on a computer.

### 3.3 Ethics

Ethical approval for this research was granted by the UCL Department of Information Studies Ethics Chair, while the study data protection was approved by the UCL Data Protection team. Informed consent was obtained from all participants before the study.

### 3.4 Pre-task questionnaires

#### 3.4.1 Demographics

This questionnaire was administered before the card game and collected demographic information such as participants’ age, gender, and living history (i.e., country of origin, country of residence, and the number of years spent in both).

#### 3.4.2 Personality variables

A self-report questionnaire was used to collect data about participants' personality (Big Five Inventory, BFI). The BFI was chosen because it is a comprehensive measure of personality characteristics and has proven to be valid and reliable across different populations (Worrell and Cross Jr, 2004; Fossati et al., 2011). BFI was derived from John and Srivastava (1999) book. Participants scored 44 statements on a 5-point Likert scale ranging from 1 – ‘I strongly disagree’ to 5 – ‘I strongly agree’. Each of the statements was related to one of the five personality dimensions – Openness, Conscientiousness, Extraversion, Agreeableness, and Neuroticism.

#### 3.4.3 Locus of control (LOC)

LOC was measured through the Internal Control Index (ICI), which was obtained from Duttweiler (1984) research. The ICI was chosen because it is empirically shown to be a reliable and more accurate measure of LOC than other questionnaires (Maltby and Cope, 1996; Meyers and Wong, 1988). Participants rated 28 statements on a 5-point scale ranging from 1 – ‘Rarely (Less than 10% of the time)’ to 5 – ‘Usually (More than 90% of the time)’. The statements related to internal control, with a low ICI score indicating external LOC and a high ICI score indicating internal LOC. Sixteen items from the BFI and 13 items from the ICI were reverse scored.

### 3.5 Card game

The task given to the participants was a game in which the player had to identify the pattern in a sequence of five cards and decide whether the value of the last card, covered, was higher or lower than the fourth card in the sequence. The decision made had no consequences, beyond entertainment. The participants had ten trials at deciding the number on the last card in the given sequence of five cards (four uncovered cards, one covered). A card could have had a number from 0 to 9. Each number was generated at random as such it could have recurred in any of the following cards (e.g. a sequence such as 2,4,2,4 ?, was possible, as well as 4,4,4,4,?). A participant's decision on the value of the last card could have been made both considering only the last occurring card, or the whole pattern presented.

**Figure 3:**
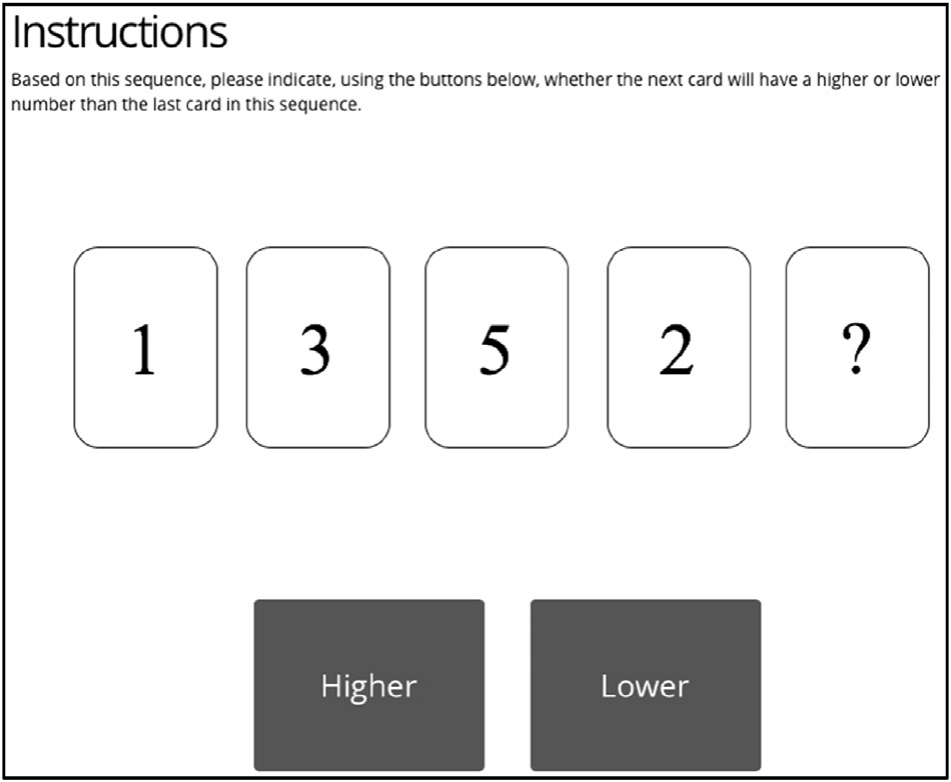
Practice trial Instructions.

In the human and AI conditions, participants received a suggestion. The suggestion was presented in the form of an arrow indicating either the ‘Higher’ or the ‘Lower’ buttons. Figure 3 illustrates the card game. The suggestion had been generated at random, to prevent it from occluding the effects of personality and LOC on trust. After making a choice (pressing the higher or lower button), all participants received visual feedback, indicating whether their choice was correct or incorrect (protocol similar to Chancey et al. (2015)). Again, there was no pattern, and the feedback was randomised.

**Figure 4:**
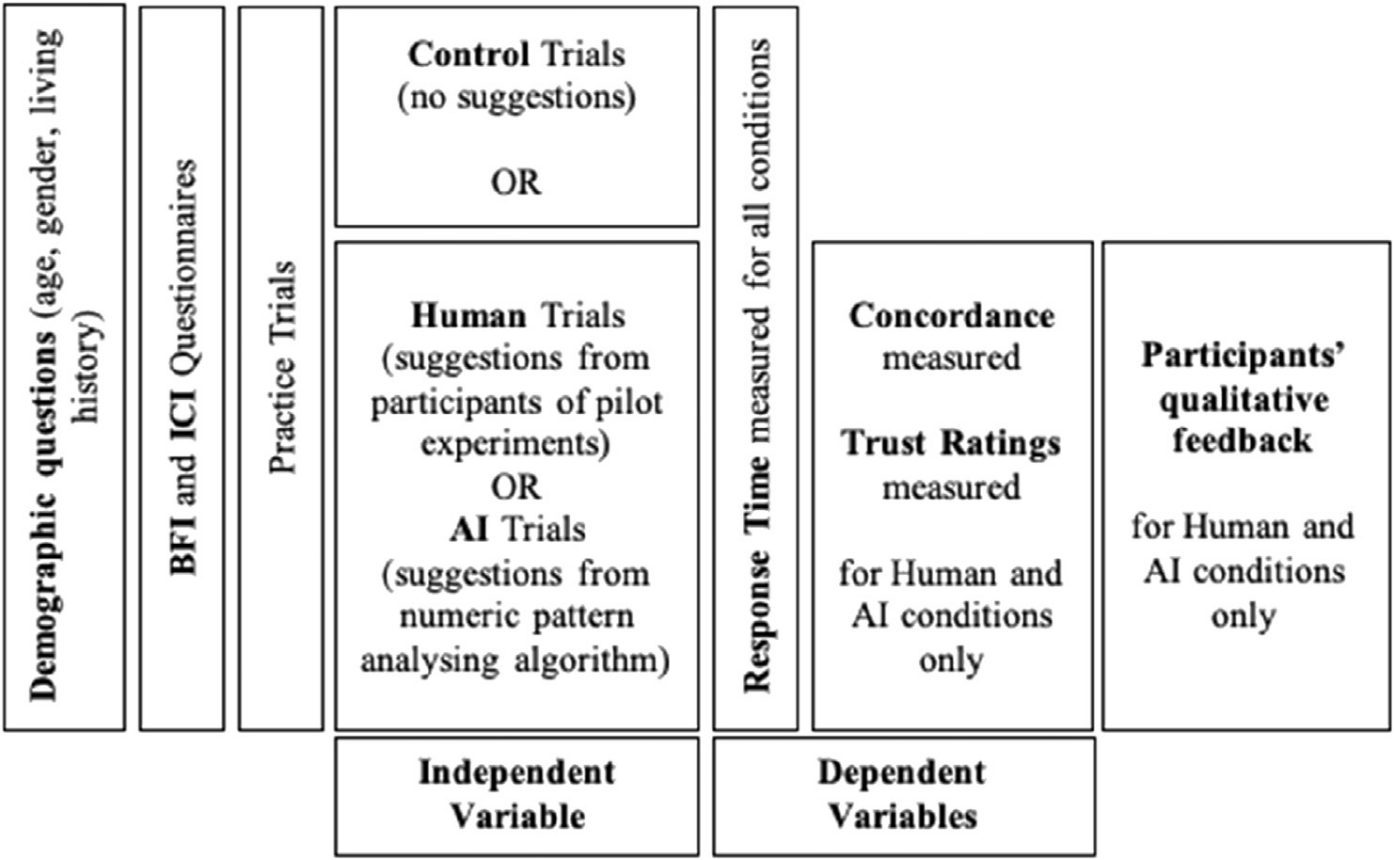
Design and procedure flow.

Participants were not aware of how the suggestions were generated and, depending on the condition, they were told that the suggestions were either created by the participants from the pilot study or generated from an AI algorithm analysing the numeric pattern. All participants completed the same three practice trials (see Figure 4) before the experimental trials to understand the interface of the game, where they did not receive any suggestions or feedback.

### 3.6 Post-task questionnaire

After the game, participants in the human and AI conditions were asked to report how much they trusted the suggestions on a 5-point scale ranging from 1 – ‘I did not trust the suggestions at all’ to 5 – ‘I completely trusted the suggestions’. They were also asked to explain, in a few written sentences, their rating and their reasoning behind trusting or not trusting the suggestions.

### 3.7 Design

The independent variable was the agent delivering the suggestion in three conditions – humans, AI, and the control condition with no suggestions. A between-samples design was used, and participants were randomly assigned to only one of the conditions.

The dependent variables were *concordance* - the number of participants’ responses that were the same as the suggestion, *reaction time* (RT) - the time taken from when the card sequence screen appears to when the participant clicks on either higher or lower button and the self-reported *trust ratings* - the extent to which they rated they rusted the given suggestions. The personality variables or LOC were not controlled. The personality traits and LOC mean, and standard deviation, are reported in Table 2 below. Participants were randomly assigned to one of the three conditions, and the experiment was double-blind. Figure 4 illustrates the study design and procedure.

**Table 2:** Pearson correlations, Mean and Standard Deviation.

	Mean	Std. Deviation	(1)	(2)	(3)	(4)	(5)	(6)	(7)	(8)
(1) Openness	34.46	5.39								
(2)Conscientiousness	30.44	5.05	0.048							
(3) Extraversion	23.81	6.24	.212∗	0.139						
(4) Agreeableness	33.27	5.27	0.028	0.138	.247∗∗					
(5) Neuroticism	26.06	6.02	0.027	-0.081	.322∗∗	.475∗∗				
(6) LOC	95.60	11.47	.208∗	.345∗∗	.364∗∗	0.128	.353∗∗			
(7) Reaction Time	12.37	10.26	0.159	0.025	-0.011	0.09	-0.125	0.145		
(8) Concordance	4.80	1.36	0.062	-0.127	-0.029	0.172	-0.165	-0.17	0.054	
(9) Trust Ratings	2.35	0.95	0.019	0.022	0.113	0.055	-0.162	0.101	0.074	.220∗

∗ significance at the .05 level (2-tailed); ∗∗significance at the .01 level (2-tailed).

## 4 Results

Reaction time was measured in milliseconds but is reported here in seconds. Concordance ranged from 0 - when participants did not follow the suggestion on any trial, to 10 - when participants followed the suggestions every trial. Self-reported trust ratings ranged from 1 - when participants did not trust the suggestions at all, to 5 - when participants completely trusted the suggestions.

### 4.1 H1 & H2 influence of personality traits and LOC on trust

The Big Five Inventory (BFI) was used to measure different aspects of the participants' personalities, and LOC was measured with the Internal Control Index (ICI).

Table 2 illustrates the correlation matrix amongst all the variables. The BFI variables do not correlate with the trust variables. However, amongst the measurements of trust, there is a significant positive correlation between trust ratings and concordance. Also, there is a significant positive correlation between LOC all the personality traits, excluding agreeableness.

#### 4.1.1 Reaction time

Hierarchical multiple regression was conducted to determine if the BFI dimensions and LOC predicted variance on the trust variables; the results are reported in Table 3. In step 1, the BFI dimensions did not significantly predict reaction time. In Step 2, when considering the BFI dimensions and LOC again, they did not significantly predict reaction time. Interestingly, when considered together none of the BFI dimensions beta weights were not statistically significant, except for openness (Step 1, Beta = 0.189, p = .055; Step 2, Beta = 0.17, p = .09). A simple linear regression was carried out considering only openness as a predictor variable for reaction time and found mildly significant *F*(1, 111) = 2.889, *p* = .092, *R*^*2*^ = .025, that is 2.5% of the reaction time variance can be explained by openness when considering together the conditions Humans and AI. Considering the human condition and the AI condition data separately, a simple linear regression indicated that openness was a predictor variable of reaction time for the humans condition, *F*(1, 55) = 3.205, *p* = .079, *R*^*2*^ = .055, Beta = .235. While, when considering the AI condition, openness was not a significant predictor.

**Table 3:** Hierarchical regression analysis examining the predictive validity of BFI and LOC on reaction time.

	b	Beta	t	Sig.
**Step 1**				

Openness	0.189	0.189	1.938	0.055∗∗
Conscientiousness	0.008	0.008	0.088	0.93
Extraversion	-0.115	-0.115	-1.107	0.271
Agreeableness	0.031	0.031	0.284	0.777
Neuroticism	-0.152	-0.152	-1.363	0.176

**Step 2**				

Openness	0.17	0.17	1.709	0.09∗∗
Conscientiousness	-0.023	-0.023	-0.229	0.819
Extraversion	-0.137	-0.137	-1.292	0.199
Agreeableness	0.047	0.047	0.428	0.669
Neuroticism	-0.115	-0.115	-0.972	0.333
LOC	0.11	0.11	0.966	0.336

∗∗ significance at the 0.01 level (2-tailed).

#### 4.1.2 Trust ratings

Table 4 illustrates a hierarchical multiple regression was conducted to determine if the BFI dimensions, and LOC, predicted variance on trust ratings. In Step 1, the regression considering the BFI was not significant, and Hypothesis 1 was rejected. In Step 2, LOC was added to the BFI dimensions, and the regression was again not significant. However, the beta coefficients at Step 2 for Neuroticism (Step 2, Beta = - .253, *p* = .032) and LOC (Step 2, Beta = -.274, *p* = .002) were significant, in a negative direction. Multiple linear regression was carried out considering only Neuroticism and LOC as predictor variables for trust ratings and found significant, *F*(2, 109) = 3.371 *p* = .038, *R*^*2*^ = .058, where the coefficients are significant in a negative direction (neuroticism Beta = - .229, *p* = .023; LOC Beta = - .016, *p* = .057), indicating that as levels of LOC and neuroticism increase, self-reported trust ratings decrease.

**Table 4:** Hierarchical regression analysis examining the predictive validity of BFI and LOC on trust ratings.

	b	Beta	t	Sig.
**Step 1**				

Openness	0.002	0.012	0.117	0.907
Conscientiousness	0	0.001	0.014	0.989
Extraversion	0.01	0.066	0.632	0.529
Agreeableness	-0.008	-0.042	-0.384	0.702
Neuroticism	-0.025	-0.161	-1.426	0.157

**Step 2**				

Openness	0.01	0.059	0.599	0.55
Conscientiousness	0.015	0.08	0.803	0.424
Extraversion	0.019	0.123	1.167	0.246
Agreeableness	-0.015	-0.082	-0.76	0.449
Neuroticism	-0.04	-0.253	-2.175	0.032∗
LOC	-0.023	-0.274	-2.438	0.016∗

∗ significance at the 0.05 level (2-tailed).

#### 4.1.3 Concordance

**Figure 5:**
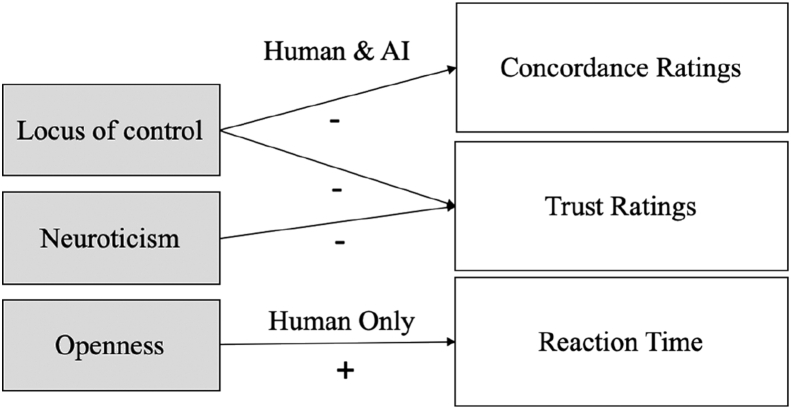
Visual representation of the linear regression results.

Table 5 llustrates a hierarchical multiple regression, which was conducted to determine if the BFI dimensions and LOC predicted variance on concordance. In Step 1, BFI was not significant, and Hypothesis 1 was rejected for concordance. In Step 2, LOC accounted for a mild significant variance beyond the BFI dimensions *F*(6, 111) = 1.889, *p* = .090, *R*^*2*^ = .097. As such, Hypothesis 2 was supported for concordance. Interestingly, LOC (Beta = -0.196, *p* = .083) was statistically significant but in a negative direction. That is, as LOC decreased, concordance increased, and vice versa, beyond the BFI dimensions. Considering only LOC as a predictor variable for concordance, a simple linear regression was found significant, *F*(1, 111) = 3.291 *p* = .083, *R*^*2*^ = .029, Beta = -.170 (see Figure 5).

**Table 5:** Hierarchical regression analysis examining the predictive validity of BFI and LOC on concordance.

	b	Beta	t	Sig.
Step 1				

Openness	-0.011	-0.045	-0.462	0.645
Conscientiousness	-0.037	-0.138	-1.455	0.149
Extraversion	-0.016	-0.071	-0.695	0.489
Agreeableness	0.043	0.164	1.524	0.131
Neuroticism	-0.027	-0.12	-1.089	0.278

Step 2				

Openness	-0.003	-0.011	-0.111	0.912
Conscientiousness	-0.022	-0.082	-0.822	0.413
Extraversion	-0.007	-0.031	-0.298	0.766
Agreeableness	0.035	0.135	1.253	0.213
Neuroticism	-0.042	-0.186	-1.612	0.11
LOC	-0.024	-0.196	-1.753	0.083∗∗

∗∗ significance at the 0.01 level (2-tailed).

### 4.2 H3: algorithm appreciation

#### 4.2.1 Reaction time

Participants responded fastest in the AI condition, followed by the humans, and control conditions. Mean reaction time was lowest in the AI condition (*n* = 56, *M* = 11.56s, *SE* = 1.39s) followed by the human condition (*n* = 57, *M* = 13.06s, *SE* = 1.32s) and the control condition (*n* = 58, *M* = 19.27s, *SE* = 5.61s). A significant Levene test (*F*(2,169) = 6.49, *p* = .002) indicated a skewed distribution of variance for RT. A Kruskal-Wallis test showed that there was no overall effect of condition on RT (χ^2^(2) = 4.292, *p* = .117). A Mann-Whitney test only showed a significant difference between the control and humans conditions (*U* = 1290, *p* = .021). No significant differences were found between the control and AI (*U* = 1535, *p* = .255) and the human and AI (*U* = 1384, *p* = .087) conditions.

#### 4.2.2 Concordance and trust ratings

Mean trust ratings were significantly higher in the AI (*M* = 2.53, *SE* = 0.13) than humans (*M* = 2.16, *SE* = 0.12) condition (*t*(112) = -2.11, *p* = .019, $\eta_{\rho }^{2}$= 0.04). Also, an independent samples *t*-test showed a significant difference between humans (*M* = 4.58, *SE* = 0.15) and AI (*M* = 5.02, *SE* = 0.20) conditions for concordance (*t*(112) = -1.74, *p* = .043, $\eta_{\rho }^{2}$= 0.26).

#### 4.2.3 Relationship between concordance and trust ratings

The trust ratings were self-reported, whereas the concordance was empirically measured. These two variables together provided a holistic measure of trust. Therefore, there would be a relationship between concordance and trust ratings. The median trust rating (*Mdn* = 2) was used to categorise concordance into high and low trust rating groups. In the AI condition, a significant difference was found between the high trust rating group (*n* = 27, *M* = 4.59, *SE* = 0.29) and the low one (*n* = 29, *M* = 5.40, *SE* = 0.26) (*t*(55) = 2.08, *p* = .0215, $\eta_{\rho }^{2}$= 0.07). There was no significant difference between the concordance of low and high trust rating groups in the human condition.

## 5 Discussion

The primary research question is to what extent personality traits and locus of control influence trust formation in the context of a game-based decision-making task. Trust is measured with behavioural (reaction time, concordance) and self-reported variables (trust ratings). The research also investigated the effect of the agent delivering a suggestion (human or AI) on reaction time, concordance and trust ratings.

### 5.1 Trust behaviour & LOC

Our findings are novel with regards to Locus of Control and trust behaviour (measured through concordance). LOC as a negative predictor of concordance ratings indicates that people with an internal LOC, as described in the literature, are more self-reliant, experience a greater need to be in control of their decision and concord less with the suggestion. While those with an external LOC, described as more consultative in literature, tend to follow suggestions more. This behaviour was observed for both suggestions from humans and AI. The findings are especially new in the context of AI-based algorithms and have implications for the design of decision-aiding AI-based systems, as will be discussed in the section below. The task used in this experiment did not have any consequences and was not under time pressure. This means that participants were not persuaded to follow the suggestion either by AI or other people or due to time. Despite that, participants with low LOC still showed patterns of concordance with humans and AI suggestions.

### 5.2 Trust beliefs, LOC and neuroticism

Considering trust beliefs (as seen through self-reported trust ratings), in light of the results of this study, it can be argued that people's personality factors (neuroticism and LOC) override any external influences on trust. These findings provide insight into the extent to which people are selective in whom they trust for information and how individual and personality traits support such selectivity. The following comment from one of the participants supports this reasoning.

‘*I evaluated the sequence of numbers myself and thought of the answer before I looked at the suggestion. If it lined up with my idea, then I chose the answer I thought of, if it didn't, I thought about it again, but stayed with my own decision most of the time.*’ (male, age 19, AI, internal LOC).

It is known that those who have a high internal LOC attribute their successes and failures to their own decisions (Joelson, 2017), as such it is possible that participants with an internal LOC did not trust the suggestions as a defensive mechanism to preserve their self-esteem. For example, if they followed the suggestion instead of their own judgement and received negative feedback, they would attribute this failure to a wrong decision made by them (in trusting the suggestion) instead of an inaccurate suggestion, and that would affect their self-esteem.

In addition, high neuroticism decreases self-reported trust ratings both when suggestions come from humans or AI. This is only in line with their tendency to experience negative emotions (John et al., 2008) and most likely be more negative in their perceptions. Fahr and Irlenbusch (2008) and Müller, J., and Schwieren, C. (2019) used the BFI traits (measured with different scales) to analyse trust behaviour amongst humans in an organisation utilising a trust game and found that anxiety is linked to distrust amongst humans. The present findings extend this result also when a suggestion comes from an AI-based algorithm.

Interestingly, neuroticism is not a predictor of concordance, although correlated with self-reported trust ratings. As such, it can be speculated that people with high neuroticism perceived their trust to be low. However, this did not manifest itself in their concordance behaviour with the suggestions provided, whether the suggestion came from humans or AI.

### 5.3 Openness & trust of humans

According to Simpson (2012), humans live socially, and trust is rooted in their need to cooperate. For the human condition only (interpersonal trust), openness was found to be a predictor of reaction time. In particular, people were faster in their response when the openness trait had a high value, in line with the open-mindedness of their personality. The interpersonal trust literature (Thielmann and Hilbig, 2015; Mooradian et al., 2006; Jacques et al., 2009) suggests that agreeableness should have an effect on interpersonal trust; also, Jacques et al. (2009) suggest extraversion to be positively correlated with trusting behaviour. Such results were not found in this study. However, it should be noted that the above findings are based on a systematic review of the literature, while in the current study, the hypotheses were actually put at test. The findings are novel, and as Alarcon and colleagues (2018) state, there is a scarcity of research investigating specific personality traits across the trust process.

### 5.4 Do we trust humans more or AI?

Finally, this study also considered whether people trusted more other humans or the AI-based algorithm. The results show that whether the suggestions come from previous participants or an AI made a difference in the trust ratings. This is in line with Lewandowsky et al. (2000) results, indicating that people trust automation more than humans, and especially when they cannot rely on their own judgement.

The number of people that reported trusting themselves more than the suggestion was higher in the human condition, where people in their comments, were more dismissive of the suggestion from previous participants stating that their trust was reduced by the fact they did not know them, and they did not repute them experts. This is in line with the literature, as Logg et al. (2019) suggest that people undervalue the advice of others, while they rely more on the one coming from an algorithm. Also, Madhavan and Wiegmann (2004) found that automation trust is linked to performance, while for humans is linked to dispositional characteristics (e.g. effort, expertise). Interestingly, participants also reported in their comments that if an explanation on the reasoning behind the suggestion were provided, they would have trusted it more, which is in line with Verberne et al. (2012) who found that trust in humans, as for an AI, depends on sharing information and goals when taking over a task.

When considering reaction time, it was found that participants incorporated suggestions and responded fastest in the AI, followed by the human and control conditions. However, the differences in reaction time between the conditions were not significant. The skewed distribution of variance for RT and the high standard error for the control condition could explain why the differences were not significant.

Concordance and trust ratings were significantly higher for the AI than the human condition. This illustrates that people trust AI more than other people and supports the idea of algorithm appreciation (Lewandowsky et al., 2000; Logg et al., 2019). Furthermore, in the AI condition, there was a significant relationship between self-reported trust ratings and empirically measured concordance, suggesting that participants were consciously aware that they were following the suggestions and attributed this to trust in the suggestions.

### 5.5 Theoretical implications

This study has implications for personality psychology and research related to trust. Evans and Revelle (2008) proposed that individual differences could predict trusting behaviour. In other words, trust is an enduring trait rather than a transient state and relates to people's underlying individual differences. The authors suggest that dimensions such as neuroticism relate to one's willingness to accept vulnerability. The authors continue stating that individual differences influence attraction to rewards and sensitivity to punishment, which in turn influence trust propensity. The results of this study support the idea that neurotic participants and those with an internal locus of control trusted human suggestions less to avoid putting themselves in a vulnerable position, relying more on their own judgment. Thus, present findings support the idea that trust is a compound trait that affects behaviour, and therefore, a more complex view of personality needs to be adopted, one in which trust is seen as a trait that relates not only to underlying behaviour, but also other personality traits.

### 5.6 Implications for the development of AI-based technologies

When considering trust in an AI-based algorithm, our results indicate that people's personality traits (neuroticism and LOC) might override other factors, suggesting that for AI-based decision support systems to be trusted, their design might have to consider people's personality traits. While it is difficult to control for all effects of personality on trust, perhaps if users are given the option to customise some of the algorithmic output, in a manner that makes them feel less vulnerable, more in control and less anxious, it may increase trust.

Some of the participants commented that they did not know how the algorithm functioned, and this led to distrust. This appears to suggest that unless users understand correctly how an algorithm produces an output, they will be unable to trust the algorithm. Thus, one possible way to increase trust in AI could be through increasing transparency and explaining to users how an AI algorithm works, thereby furthering the case for explainable AI to make the AI reasoning transparent (Holzinger et al., 2017). However, present findings suggest that the need for explainability goes beyond the current efforts in explainable AI. There might be a need to considers human traits in AI technology development, which have not been explored in this study. It is suggested that AI-based decision support systems should be designed with a focus on people (i.e., with a basis in psychology and cognitive science) rather than only considering the best AI technology for the problem, as also suggested by Miller et al. (2017). Research showed that (Yang et al., 2017) increased transparency reduces the ‘cry wolf’ effect, which is when the threshold to trigger an alarm is set low and repeated false alarms cause users to lose trust in the system. More transparency would allow users to identify false alarms without losing trust in the system. This is also supported by the comments of some participants on the suggestions from AI.

‘*Even though [the suggestion] has a probability attached to it, it's still not enough for me to trust [it].*’ (female, age 20, AI, internal LOC)

### 5.7 Limitations and alternative explanations

There were a couple of notable limitations associated with the experimental design. First, the card game was chosen to prevent any effect of expertise, have no consequences or time pressure. However, the game was also meaningless and making a wrong judgement did not have any consequences. In real life, advice from another person or an AI, if used to make serious decisions (e.g., medical diagnoses) could potentially have devastating consequences. As such, the nature of the task, or the context could have influenced the extent to which participants trusted the suggestion. From the literature, it is known that an antecedent of trust is also environmental-based factors (Sanders et al. (2011)), and task context (Schaefer et al., 2016). As such, in further research to better understand how trust in AI develops, an actual decision support system could be used, in which participants would have to make decisions with tangible consequences.

Participants were suggested, in both the humans and AI condition, to answer higher on half the trials and lower on the other half. Still, the suggestions were randomised to prevent participants from suspecting this. However, several participants reported in the feedback that the suggestions seemed arbitrary to them. This could have reduced participants' trust in the suggestions and confounded the results.

‘*I think the suggestions were arbitrary and I would trust my own judgement more.*’ (female, age 18, Human, internal LOC)

Furthermore, in the human condition, participants were told that the suggestions were from previous participants from a pilot study. Some participants reported that they did not trust the suggestions because they did not know anything about the pilot participants' performance, and their suggestions could be wrong or misleading. This could have confounded the results as participants' lack of trust in this condition may not be because the suggestions are by humans but because they do not have enough information to trust the suggestions.

‘*No basis of trusting them because previous participants do not have more knowledge about the answers than I do.*’ (age 19, female, Human, internal LOC)

In addition, participants were asked to provide their trust ratings after all the trials, and they may have only remembered the last few trials and based their ratings on the same. Participants were given feedback after every trial, which was positive (i.e., told that their response was correct) for half the trials and negative (i.e., told that their response was incorrect) for the other half. While this was randomised, it is possible that participants answered in line with the suggestion and received a stream of positive feedback toward the end of the experiment, which could have increased the final trust ratings. Alternatively, they could have received negative feedback, which could have decreased trust. The effect of feedback and participants' experience with the agent is unknown, and it could have influenced the development of trust and by extension, the results.

‘*I had a few wrong answers after trusting the suggestions, so it became less trustworthy.*’ (age 22, female, AI)

Finally, personality data were collected using self-report questionnaires. Self-reports reflect how participants view themselves as opposed to their actual state of nature, and so, the data obtained may be biased. Using empirical measures for personality would have yielded more accurate data.

### 5.8 Future research

In line with one of the drawbacks of this study, future research could investigate trust with real-life decision support systems and incentive (e.g., awarding money for right decisions and deducting money for wrong decisions) could be used to ensure that participants take the task seriously. As outlined in one of the previous sections, this research found a new and exciting relationship between LOC neuroticism and trust beliefs. This could be studied further, specifically in terms of how LOC influences interpersonal trust and relates to other personality traits like those in the BFI. Qualitative research (e.g., interviews) could investigate the motives behind the choices made by participants, to aid the understanding of the type of explanation (in terms of explainable AI) would be required by which personality type and LOC.

Age and the country in which the participants spend most of their life were measured in this study. Future research could focus on how patterns of trust are influenced by these variables and the interaction between these variables. Furthermore, being an expert or well-versed in fields like AI, computer science, robotics, human-machine communication, etc. could influence trust formation and should be studied further. This study could also inform a second study to validate the results found here. A study (Stokes et al., 2010) examined the effect of mood on trust in technology by inducing participants into a positive or negative mood before making them complete a computer-based task using an automated aid. They found a significant effect of mood on initial trust formation. With a negative mood, trust was significantly less than with a positive mood. However, this effect decreased as interaction with the aid increased. It is known that people rely on trust when they cannot make rational choices, which suggests that trust has an affective component. The long-term effects of mood during initial trust formation on trust in AI and quality of subsequent judgements made with the help of AI could be investigated.

As mentioned earlier, this study shows that interpersonal trust is different from trust in technology; however, the AI was described as an algorithm, and the numeric suggestions did not lead to anthropomorphic interpretations. Automation anthropomorphism, which is the degree to which automation is human-like, has been associated with greater trust resilience (de Visser et al., 2016). In other words, trust in anthropomorphised automation is less likely to breakdown than trust in traditional algorithms. However, there may also be good reasons to decrease or exclude anthropomorphism in the design of automated agents, simply because human features may be unnecessary or even damaging. Future research could investigate under what conditions are automation anthropomorphism needed, and the quantitative and qualitative differences in trust in AI when it is presented to users as an intelligent algorithm, whereas when it is presented with human-like qualities.

## 6 Conclusion

The overall aim of this study was to investigate the effect of personality and locus of control on interpersonal trust; it is interesting to observe that personality traits influence trust. Thus, it is suggested that trust is a compound trait that affects behaviour, and a more complex view of personality needs to be adopted that includes trust. These findings indicate that personality often overrides any external influence on trust. LOC is a factor in reaction time, and participants with high an internal LOC took longer to respond; however, they also agreed less with the suggestion. Participants with high neuroticism and extraversion have lower concordance ratings than participants with low scores in these traits.

The extent to which people trust suggestions delivered by AI and humans in a game decision-making task and how individual and personality factors influence trust formation was also investigated. Participants reported trusting AI more than humans, and incorporated suggestions from AI more than humans (as observed through concordance).

Finally, trust is a crucial topic for the development of future technologies, as distrust toward AI could hinder the development of intelligent systems that improve performance in decision-making tasks. From a human-machine interaction perspective, there is a need to understand the extent to which people trust AI-based algorithms and how this could be improved.

## Declarations

### Author contribution statement

D. M. Romano: Conceived and designed the experiments; Performed the experiments; Analyzed and interpreted the data; Contributed reagents, materials, analysis tools or data; Wrote the paper.

N. N. Sharan: Conceived and designed the experiments; Analyzed and interpreted the data; Contributed reagents, materials, analysis tools or data; Wrote the paper.

### Funding statement

This research did not receive any specific grant from funding agencies in the public, commercial, or not-for-profit sectors.

### Competing interest statement

The authors declare no conflict of interest.

### Additional information

No additional information is available for this paper.
